# The accuracy of delirium assessment by cardiologists treating heart failure inpatients: a single center retrospective survey

**DOI:** 10.1186/s13030-020-00188-6

**Published:** 2020-07-29

**Authors:** Anna Hayashi, Sayaka Kobayashi, Kentaro Matsui, Rie Akaho, Katsuji Nishimura

**Affiliations:** 1 Department of Psychiatry, Tokyo Women’s Medical University, 8-1, Kawada-cho 8-1, Shinjuku-ku, Tokyo, Japan; 2 Department of Psychiatry, Saitama Medical Center, Saitama Medical University, Saitama, Japan; 3 Department of Sleep-Wake Disorders, National Institute of Mental Health, National Center of Neurology & Psychiatry, Tokyo, Japan; 4 Department of Clinical Laboratory, National Institute of Mental Health, National Center of Neurology & Psychiatry, Tokyo, Japan

**Keywords:** Delirium, Heart failure, Consultation-liaison psychiatry

## Abstract

**Background:**

Patients with heart failure (HF) accompanied by delirium are at risk of rehospitalization and death, thus early detection and appropriate treatment is imperative. Palliative care for patients with HF is an important issue, particularly for patients who also have delirium. This retrospective study examined the accuracy of delirium assessment by cardiologists treating patients with HF, identified factors related to the detection of delirium, and recorded the initial treatment.

**Methods:**

This was a retrospective chart survey of 165 patients with HF referred to a consultation liaison (C-L) service during treatment in the cardiology wards of a general hospital over a 6-year period. Diagnosis of delirium by the C-L psychiatrists was based on DSM-IV-TR.

Cases in which cardiologists had stated “delirium” in the medical records were classified as an accurate assessment of delirium (Agreement group). Cases in which cardiologists did not state “delirium” were classified as Disagreement.

**Results:**

Among 81 patients with delirium (51 [62.9%] male; 74.7 ± 13.3 years old), the ratio of accurate assessment of delirium by cardiologists was 50.6% (*n* = 41; Agreement group). Age, sex, and HF severity did not differ significantly between the two groups. Although disquietedness was identified most frequently (*n* = 59, 73%), only 33 of these 59 patients (55.9%) were recognized as having delirium by cardiologists.

Inappropriate initial treatments were only noted in the Disagreement group. In both groups, most cases were referred to a C-L service without new medication for psychiatric symptoms.

**Conclusions:**

An accurate assessment of the delirium of inpatients with HF by cardiologists was found in only around half of all cases. Accurate detection is important to avoid harmful drug administration and to provide appropriate palliative care.

## Background

Heart disease is the second most common cause of death, following cancer, in Japan. Because heart failure (HF) is the terminal condition of all heart disease, it is a common cause of death among patients with heart disease. A total of 260,000 patients with HF were hospitalized in 2016, and this number has increased by more than 10,000 cases annually over the past 6 years in Japan [1]. It is also known that HF is associated with a substantial financial burden for medical care [2]. Thus, the management of HF is an important issue.

Delirium can be caused by serious physical disorders and is characterized by an acute disturbance in attention and cognition [3]. The frequency of delirium in patients with HF has been reported to be 25–75% [4]. Delirium in HF inpatients is associated with a variety of risks, including rehospitalization and death [5]. In addition, delirium in patients with acute decompensated HF has been associated with a worsening of HF and a prolonged hospital stay [6]. Previous studies have also reported that delirium increases hospital costs [7, 8]. Thus, palliative care for HF is an increasingly important issue [9], particularly in patients who also have delirium.

Early detection of delirium and appropriate treatment are strongly recommended in the clinical practice guideline published by the Society of Critical Care Medicine [10] and are also regarded as important in the care of patients with HF [11]. Chief physicians are typically the first to notice delirium in inpatients and to pursue treatment. Thus, appropriate management of delirium in patients with HF by cardiologists is particularly important. However, approximately half the number of cases of cognitive dysfunction, including delirium, are overlooked and untreated in patients with HF [12]. If the cause of problematic behavior in patients is misdiagnosed as another psychiatric disease, patients are likely to be treated with benzodiazepines or opioids, which are risk factors for the development of delirium [13–15]. It has been shown that delirium is improved by appropriate drug administration [16]. To manage delirium appropriately, it is critical that it is accurately assessed by cardiologists.

It can be difficult for non-psychiatrists to accurately assess various mental disorders in inpatients. The accuracy of non-psychiatrist assessments of psychiatric problems has been reported in elderly patients [17] and in patients with cancer [18]. In these studies, psychiatric symptoms, such as disquietedness or confusion, identified by chief physicians were considered to indicate delirium. However, it is unclear whether chief physicians were able to accurately attribute these symptoms to delirium. The possibility of over estimation of delirium cannot be excluded.

To the best of our knowledge, no previous studies have examined the accuracy of assessments of delirium made by cardiologists. As some previous studies have suggested in other areas, such as in patients with cancer and older populations [17, 18], it may be difficult for non-psychiatrists to identify delirium, and this can lead to the prescription of inappropriate medication. The purpose of the current retrospective chart survey was to determine the accuracy of delirium assessments in patients with HF by cardiologists, to identify factors related to the detection of delirium, and to clarify what drug therapies are used for delirium as an initial treatment by cardiologists.

## Methods

### Design and participants

This was a retrospective chart survey of Japanese patients with HF in cardiology wards, including the cardiac care unit of our general hospital, who were subsequently referred by their cardiologist to a consultation-liaison (C-L) psychiatry service during a 6-year period, between January 2009 and December 2014.

**Fig. 1:**
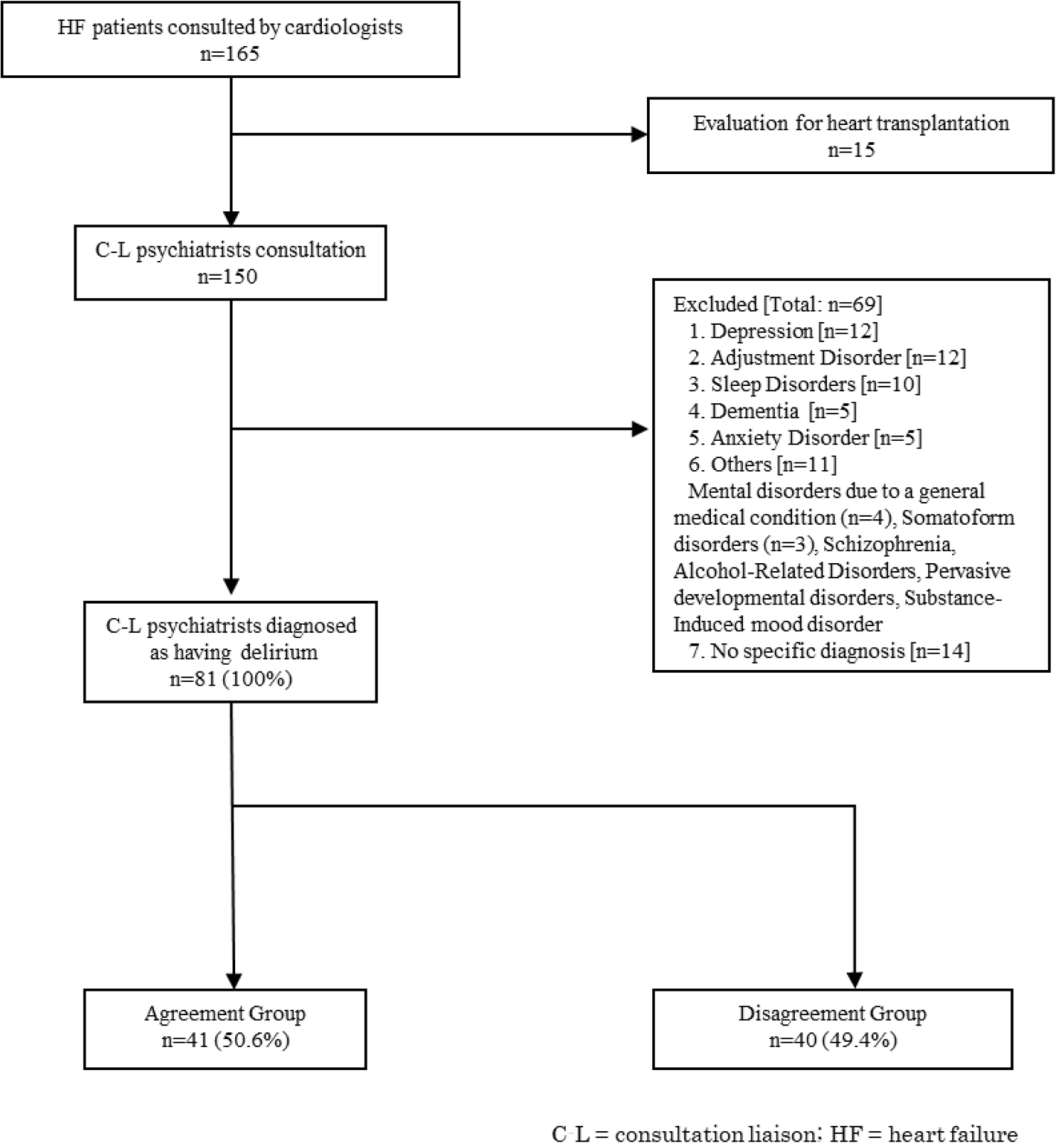
Flow diagram of this study participants

When using the C-L psychiatry service, cardiologists wrote referral letters about the purpose of the consultation and the condition of the patient. C-L psychiatrists met the patients and conducted psychiatric interviews, made a psychiatric diagnosis, and recommended treatment. Psychiatric diagnoses were based on the Diagnostic and Statistical Manual of Mental Disorders Fourth Edition: Text Revision (DSM-IV-TR) [3]. Patients diagnosed with delirium by C-L psychiatrists were enrolled as participants in the present study. The exclusion criteria were as follows: those with a psychiatric diagnosis other than delirium, those without any psychiatric diagnosis, and/or those who underwent a psychological evaluation for becoming a candidate recipient of heart transplantation (Fig. 1). Twenty-four psychiatrists were included in the C-L team during the study.

Cases in which cardiologists had explicitly written the term “delirium” in patients’ medical records or in the referral letter were classified as an accurate delirium assessment and placed in an Agreement group. Cases in which cardiologists did not use the term “delirium” in patients’ medical records or referral letters were classified as a Disagreement group. We compared the patient characteristics between the two groups to identify factors related to the detection of the delirium of HF inpatients by cardiologists. This study was approved by the Ethics Committee of the Tokyo Women’s Medical University (3771). Patients retained the right to opt out.

### Data collection and variables

We retrospectively examined referral letters and medical records from 2 days before the referral day. When the psychiatric episode had occurred earlier than 2 days before the referral, the medical records were confirmed retroactively to the time when the episode occurred. If the assessments by the cardiologists were not described using proper psychiatric terminology, we interpreted their words used to describe psychiatric symptoms based on the conventional use of psychiatric clinical terms in Japan, and carefully examined medical records to infer the intention of the referral doctors. According to the previous literature [17, 18], we classified the psychiatric symptoms described by cardiologists into categories. When we were unable to classify the symptoms into a category that had been previously used in the literature [17, 18], we created new categories. A symptom was only classified as “delirium” when cardiologists had explicitly written that term in the medical records or referral letter.

For each patient, we classified the initial treatment provided by their cardiologist before the C-L service consultation. According to the National Institute for Health and Care Excellence [14], the administration of benzodiazepine or antihistaminic drugs is a risk factor for the development of delirium. Therefore, we considered the administration of benzodiazepines or antihistamines to be an inappropriate treatment [14, 19]. In these cases, we recommended that patients stop taking these medications.

Additionally, the following data were collected: patient characteristics (including the severity of HF, the history of complications, and mental disorders).

### Statistical analysis

All analyses were carried out using SPSS statistics version 20 (SPSS Japan, Inc., Tokyo, Japan). The data did not follow a normal distribution (tested using Kolmogorov-Smirnov tests). To compare the psychosocial and medical characteristics between the Agreement Group and Disagreement Group, a Chi-square test was used for categorical variables and the Mann–Whitney U test was used for continuous variables. The threshold for significance was set at a *P*-value of < 0.05.

## Results

### Patient characteristics

During the study period, 165 patients were referred by cardiologists to the C-L psychiatrist service. Eighty-four cases were excluded due to a psychiatric diagnosis other than delirium, the absence of any psychiatric diagnosis (*n* = 14), or consultations conducted only for a psychological evaluation for heart transplantation candidate recipients (*n* = 15) (see Fig. 1). A final total of 81 patients were included in this study.

The average age of subjects was 74.7 ± 13.3 years old. Fifty-one subjects (63.0%) were men. Regarding the severity of HF, the New York Heart Association classification (NYHA) III or IV accounted for 61 subjects (75.3%) of the total sample. The average left ventricular ejection fraction (LVEF) was 36.3 ± 13.5%, brain natriuretic peptide (BNP) was 1033.0 ± 1306.2 pg/ml, and estimated glomerular filtration rate was 40.6 ± 26.3 mL/min/1.73 m^2^. In terms of comorbidities, 52 subjects (64.2%) had hypertension and 26 subjects (32.1%) had diabetes. None of the patients reported a problem with the use of alcohol or other substances (see Table 1).

**Table 1:**
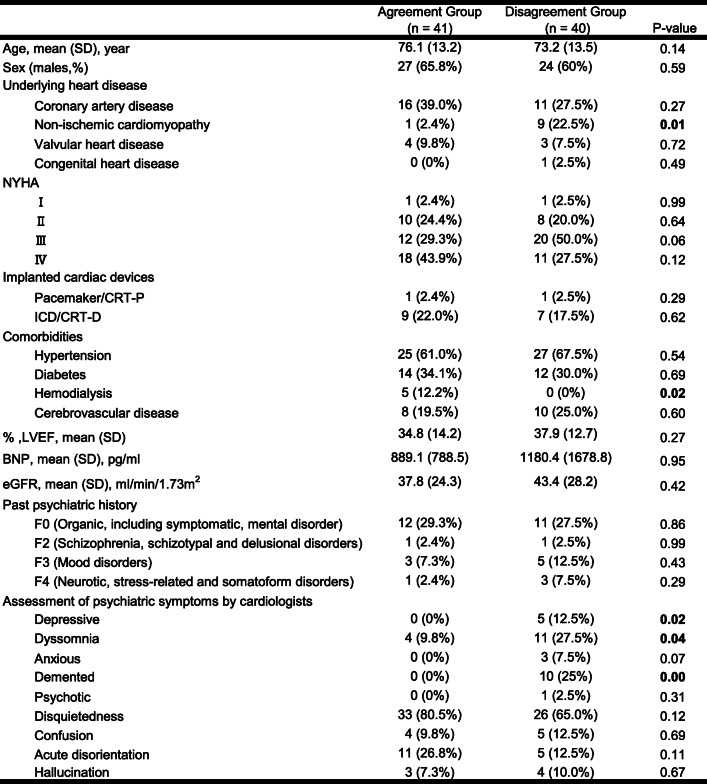
Patient characteristics

Values are shown as the .mean (standard deviation or percentage)

*BNP* Brain natriuretic peptide, *CRT-D* Cardiac resynchronization therapy defibrillator, *CRT-P* Cardiac resynchronization therapy pacemaker, *eGFR* Estimate glomerular filtration rate, *ICD* Implantable cardioverter defibrillator, *LVEF* Left .ventricular ejection fraction, *NYHA* New York Heart Association

### Agreement ratio of delirium diagnosis

Among the 81 patients who received a diagnosis of delirium by psychiatrists, cardiologists had identified 41 (50.6%) of them as having delirium (Agreement Group). The remaining 40 patients (49.4%) were misjudged as having another psychiatric disease (Disagreement Group) (see Fig. 1).

### Assessment of psychiatric symptoms by cardiologists

Psychiatric symptoms other than delirium mentioned by cardiologists were classified into nine categories. “Depressive” (*n* = 5) referred to exhibiting depressive symptoms and a lack of energy. “Dyssomnia” (*n* = 15) referred to insomnia and day-night reversal. “Anxious” (*n* = 3) referred to anxiety and anxiety-related behavioral disorders. “Demented” referred to dementia and cognitive functional disorder (*n* = 10). “Psychotic” (n = 1) referred to acute psychosis. “Disquietedness” (*n* = 59) referred to problematic behavior ranging from violent language to violence and a disquietedness. “Confusion” (*n* = 9) referred to disorganized thoughts and behavior. “Acute disorientation” (*n* = 16) referred to sudden disorientation to time, place, and/or person. “Hallucinations” (*n* = 7) referred to both visual and auditory hallucinations.

### Comparison of the two groups

We compared patient characteristics between the Agreement Group and Disagreement Group (Table 1). There was a significantly higher rate of hemodialysis in the Agreement Group than in the Disagreement Group (χ^2^ = 5.20, *p* < 0.05). There was a significantly higher rate of non-ischemic cardiomyopathy in the Disagreement Group than in the Agreement Group (χ^2^ = 7.53, *p* < 0.01). Age, sex, and severity of HF (NYHA, LVEF, and BNP) did not significantly differ between the two groups.

In the Disagreement Group, Depressive (χ^2^ = 5.46, p < 0.05), Dyssomnia (χ^2^ = 4.23, *p* < 0.05), and Demented (χ^2^ = 11.70, *p* < 0.01) categories were significantly more frequent than in the Agreement Group. While disquietedness was frequently reported for all participants with delirium (59 of 81, 72.8%), only 33 of these 59 were recognized as having delirium by cardiologists.

### Classification of initial treatment by cardiologists

In the 81 cases of delirium, initial treatments prescribed by cardiologists were as follows: benzodiazepines alone (*n* = 2), antihistamines alone (*n* = 1), antipsychotics alone (*n* = 5), dexmedetomidine alone (*n* = 3), combination of antipsychotics and dexmedetomidine (*n* = 3), combination of antipsychotics and benzodiazepines (*n* = 3), combination of antipsychotics, dexmedetomidine, and benzodiazepines (*n* = 1), and no drugs (*n* = 66). Dexmedetomidine was only prescribed in the cardiac care unit.

**Fig. 2:**
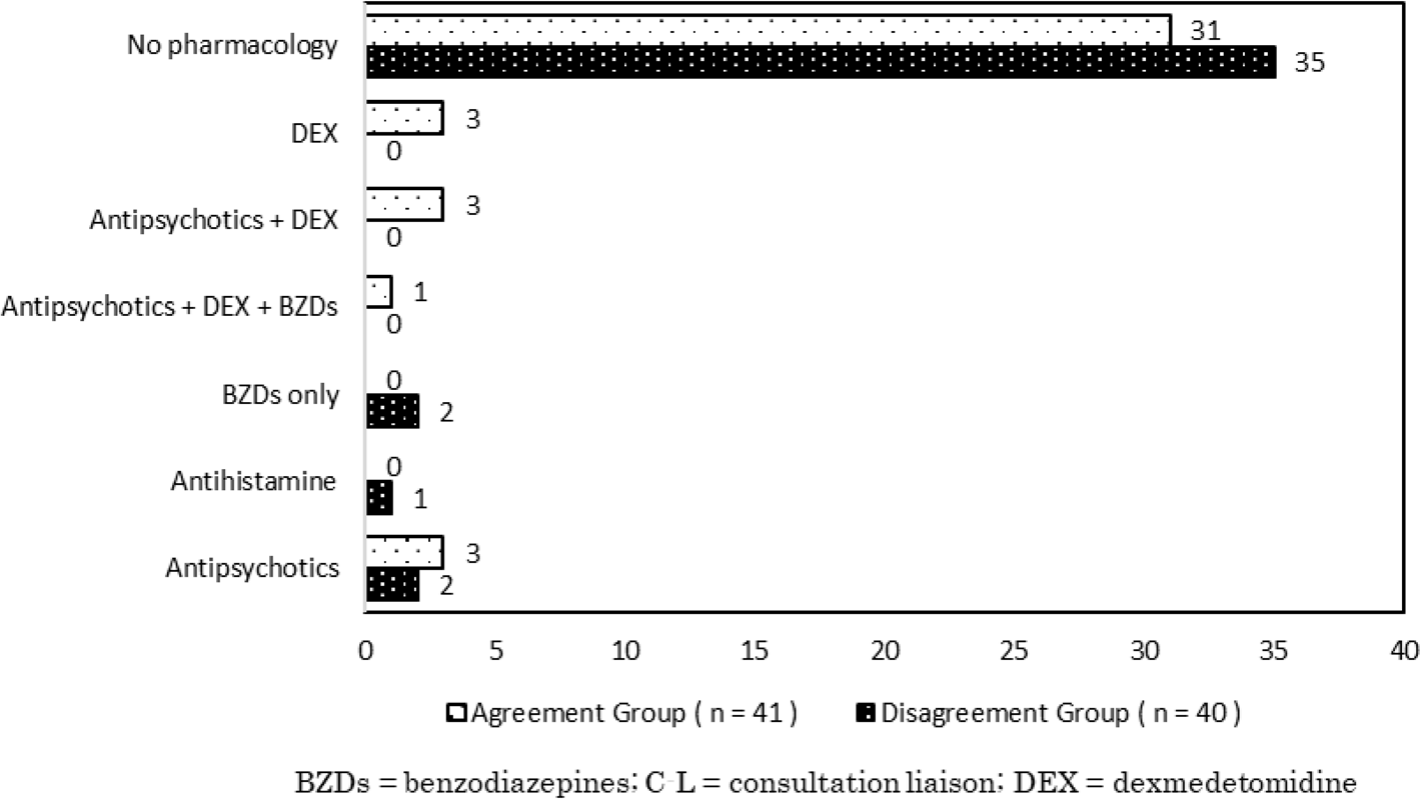
Initial Treatment by Cardiologists before C L Psychiatry Service Consultation

The between-group differences in treatments prescribed by cardiologists are shown in Fig. 2. Inappropriate treatments, such as benzodiazepines alone and antihistamines alone, were only found in the Disagreement Group. In both groups, most cases were consulted without prescribing new medication to treat psychiatric symptoms identified by a cardiologist (75.6 and 87.5%, respectively).

## Discussion

In this study, we determined the probability of a correct judgment by cardiologists of the delirium of HF inpatients. Consecutive cases referred to the C-L service over a period of 6 years were used. Among the 81 patients with delirium, only 50.6% were correctly identified by cardiologists. Inappropriate treatments for delirium were only found in the misjudged cases. Appropriate assessment of delirium by cardiologists is absolutely necessary.

The accurate assessment of delirium in HF by cardiologists occurred in approximately half of all cases. It has been reported that the correct assessment rate of delirium by non-psychiatrists is 87% in patients with cancer [18] and 69% in elderly patients [17]. In the current study, we only considered cases in which cardiologists explicitly stated “delirium” as the correct detection of delirium, which is a more precise definition of delirium than that used by previous studies [17, 18]. “Delirium” has been broadly defined in these two previous studies, including cases in which non-psychiatrists did not clearly mention delirium, such as cases in which only disquietedness was mentioned. Of the symptoms studied, “Disquietedness” was identified most frequently in both the Agreement Group and Disagreement Group. However, disquietedness (such as agitation) is not specific to delirium and can occur in other diseases, such as dementia [20]. Thus, disquietedness might not necessarily reflect delirium when assessed by non-psychiatrists.

No significant between-group differences were found in the physical factors, such as severity of HF, other than the Disagreement Group having significantly more non-ischemic cardiomyopathy patients and the Agreement Group having significantly more patients on hemodialysis. In the present study, most participants were elderly which is a direct causal factor for the development of delirium [21]. However, some potential causes of delirium, e.g., intracranial space-occupying lesions, were not fully explored in this study. We could not exclude the potential effect of these confounding factors.

Psychiatric symptoms such as “Depressive”, “Demented”, and “Dyssomnia” were mentioned by cardiologists at a significantly higher rate in the Disagreement Group in the present study. This could be due to misjudgments of the clinical manifestations of delirium to similar other psychiatric symptoms, such as depression [22], dementia [23], and insomnia [24]. It has been reported that the detection rate of delirium differs greatly between the subtypes of delirium [25, 26], and it may be easier to identify hyperactive and mixed delirium because of the obvious outward symptoms, such as aggressiveness. Hypoactive delirium is typically more difficult to detect than hyperactive delirium because its symptoms can be subtle [27]. In the current study, hypoactive delirium might have been identified as depression, dementia, or insomnia by cardiologists.

The accurate diagnosis of delirium is critical to reduce mortality and hospital stay durations by providing early and appropriate treatment [28]. In addition, if an accurate diagnosis is not obtained, delirium can worsen in the absence of the appropriate tests and treatments [22]. In the current study, there were cases of inappropriate initial treatment, such as benzodiazepines or antihistamines, in the Disagreement Group only. These findings suggest that delirium itself may have been inadvertently exacerbated as a result of cardiologists mistaking delirium as another disease [14, 15]. In contrast, these drugs were not used when delirium was accurately diagnosed by cardiologists. This result may have indicated that the chief physician had understood the proper treatment intervention for delirium. Thus, both an accurate assessment of delirium and a comprehensive understanding of appropriate therapeutic interventions are important. Although the correct diagnosis of delirium is not easy for non-psychiatrists, including cardiologists, it is essential if patients are to receive the appropriate clinical care [11]. Established screening tools such as the Confusion Assessment Method [29] can be useful for non-psychiatrists to detect delirium [14, 30]. Further studies should confirm the effect of screening tools for delirium used by cardiologists.

For the early detection and appropriate treatment of delirium in a general hospital setting, psychiatric education for non-psychiatrists is important. The present results suggest that it is essential to educate non-psychiatrists about the diagnosis of delirium, and especially about the differential diagnosis of hypoactive delirium that can include depression or cognitive dysfunction.

The current study has several limitations that should be noted. This was a small, retrospective, single-center study of Japanese patients. Participants were only the patients undergoing consultation with the C-L psychiatry service, thus not all HF inpatients were examined. The current study was a retrospective survey in a clinical setting rather than a planned prospective study, which means that the inter-rater reliability of C-L psychiatrists could not be examined. Delirium can easily be overlooked, particularly the hypoactive type, which is difficult for cardiologists to identify. The influence of the cardiologists’ characteristics and experience on their accuracy of assessment of delirium could not be examined in this study. In their diagnoses based on the DSM-IV-TR , psychiatrists did not classify delirium into subtypes. These issues should be addressed in future research.

## Conclusions

The accurate assessment by cardiologists of the delirium of patients with HF occurred in approximately half of all cases. Disquietedness is easy for non-psychiatrists to identify, but its disease specificity is low and does not lead to the correct assessment of delirium. The current findings suggest that the delirium of patients with HF is easily misdiagnosed by cardiologists as diseases with similar clinical symptoms, such as depression, dementia, and insomnia. If cardiologists were able to detect delirium more accurately, inappropriate treatment that can worsen delirium could be avoided. Thus, it is important to increase the accuracy of assessment of delirium by cardiologists.

## Data Availability

Not applicable.

## Abbreviations

HF: Heart failure
C-L: Consultation liaison
DSM-IV-TR: Diagnostic and Statistical Manual of Mental Disorders Fourth Edition: Text Revision
NYHA: New York Heart Association classification
LVEF: Left ventricular ejection fraction
BNP: Brain natriuretic peptide
